# Different expression and prognostic value of troponin in ischemic cardiomyopathy and non-ischemic dilated cardiomyopathy

**DOI:** 10.1186/s40001-023-01169-5

**Published:** 2023-07-03

**Authors:** Wei Gao, Meng Zhang, Yu Song, Xueting Han, Yamei Xu, Jingmin Zhou, Junbo Ge

**Affiliations:** 1 Department of Cardiology, Zhongshan Hospital, Fudan University, Shanghai Institute of Cardiovascular Diseases, Shanghai, China; 2 National Clinical Research Center for Interventional Medicine, Shanghai, China; 3 Department of Cardiology, Zhongshan Hospital, Fudan University (Xiamen Branch), Xiamen, China

**Keywords:** Heart failure, Ischemic cardiomyopathy, Non-ischemic dilated cardiomyopathy, Cardiac troponin

## Abstract

**Background:**

Early risk stratification of patients with ischemic cardiomyopathy (ICM) and non-ischemic dilated cardiomyopathy (NIDCM) may be beneficial for therapies.

**Methods:**

We retrospectively enrolled all patients admitted for acute heart failure (HF) between January 2019 and December 2021 in Zhongshan Hospital Fudan University, dividing them according to etiology (ICM or NIDCM). Cardiac troponin T (TNT) concentration was compared between two groups. Risk factors for positive TNT and in-hospital all-cause mortality were investigated with regression analysis.

**Results:**

A total of 1525 HF patients were enrolled, including 571 ICM and 954 NIDCM. The TNT positive patients were not different between the two groups (41.3% in ICM group vs. 37.8% in NIDCM group, P = 0.215). However, the TNT value in ICM group were significantly higher than that in NIDCM group (0.025 (0.015–0.053) vs. 0.020 (0.014–0.041), P = 0.001). NT-proBNP was independently associated with TNT in both ICM and NIDCM group. Although the in-hospital all-cause mortality did not show much difference between the two groups (1.1% vs. 1.9%, P = 0.204), the NIDCM diagnosis was associated with reduced risk of mortality after multiple adjustments (OR 0.169, 95% CI 0.040–0.718, P = 0.016). Other independent risk factors included the level of NT-proBNP (OR 8.260, 95% CI 3.168–21.533, P < 0.001), TNT (OR 8.118, 95% CI 3.205–20.562, P < 0.001), and anemia (OR 0.954, 95% CI 0.931–0.978, P < 0.001). The predictive value of TNT and NT-proBNP for all-cause mortality was similar. However, the best cutoff values of TNT for mortality were different between ICM and NIDCM groups, which were 0.113 ng/mL and 0.048 ng/mL, respectively.

**Conclusion:**

The TNT level was higher in ICM patient than that in NIDCM patients. TNT was an independent risk factor for in-hospital all-cause mortality for both ICM and NIDCM patients, although the best cutoff value was higher in ICM patients.

## Introduction

Heart failure (HF) is a growing health and economic burden globally [1, 2]. Although there are well-established therapies that help to improve prognosis, the overall 5-year mortality rate after diagnosis is approximately 50% [3]. HF can be caused by ischemic cardiomyopathy (ICM) or non-ischemic dilated cardiomyopathy (NIDCM), which can be distinguished by coronary angiography [4]. Recent studies reported that prognosis was worse in the ICM patients than that in the NIDCM patients [5, 6]. Therefore, it is important to assess the underlying HF etiology and individualize patient management.

Plasma levels of BNP or NT-proBNP are the traditional standard biomarkers and provide prognostic value for HF. In recent years, cardiac troponin has been also demonstrated to be associated with clinical outcomes in hospitalized patients with HF [7, 8]. In patients with acute decompensated heart failure, a positive cardiac troponin test was independently associated with higher in-hospital mortality [7]. However, the expression and prognostic value of cardiac troponin T (TNT) in ICM and NIDCM patients has not been fully demonstrated.

In this study, we described the expression and prognostic value of TNT in ICM and NIDCM patients, thus identified the clinical characteristics associated with TNT positive. Furthermore, we also figured out the best cutoff value in clinical application.

## Methods

### Study design and patient selection

We retrospectively screened hospitalized acute HF patients from January 2019 to December 2021 in Zhongshan Hospital Fudan University. Those patients with a discharge diagnosis of ICM or NIDCM were finally included into this study. Patients with cardiogenic shock were excluded. For ICM group, those with acute myocardial infarction were excluded. For NIDCM group, invasive coronary angiography or noninvasive coronary CT angiography were performed to eliminate significant coronary stenosis.

### Laboratory testing and echocardiography

A venous blood sample was collected for all the patients at admission. All the laboratory assays were performed by the central laboratory at our hospital. High-sensitivity cardiac troponin T (Roche Diagnostics, Switzerland) and NT-proBNP concentrations were detected at admission. Echocardiography was performed within 24 h after admission and left ventricular ejection fraction (LVEF), left atrium (LA) and left ventricular diastolic diameter (LVDD) were determined.

### Study endpoints and definitions

The main study outcome was in-hospital all-cause mortality. Those patients with TNT higher than normal reference value (0.030 ng/ml) were defined as TNT positive. For regression analysis, TNT value was divided into 4 groups (< 0.03 ng/ml, 0.03–0.3 ng/ml, 0.301–1.0 ng/ml, > 1.001 ng/ml). NT-proBNP value was divided into 5 groups (< 300 pg/ml, 301–900 pg/ml, 901–1800 pg/ml, 1801–18,000 pg/ml, > 18,000 pg/ml).

### Statistical analysis

Normally distributed data are expressed as mean ± SD, and were compared using the independent-samples T test. Skewed variables are expressed as median and inter quartile range and Mann–Whitney U test was used. Categorical data are expressed as number (percentage) and were compared using the chi-squared test. TNT and NT-proBNP were log transformed for linear correlation analysis. Logistic regression analysis was used to evaluate risk factors for TNT positive and in-hospital all-cause mortality. The included variables were common clinical factors, such as age, sex, complicating diseases, anemia, renal function and cardiac function. Receiver operating characteristic (ROC) curve and area under the curve (AUC) were used to evaluate predictive value of TNT and NT-proBNP for in-hospital all-cause mortality. All statistical analyses were performed using SPSS 22.0 (SSPS Inc., Chicago, IL, USA). A value of P < 0.05 was considered as statistically significant.

## Results

### Characteristics of included patients

A total of 1525 patients were included into this study and their characteristics were summarized in Table 1. They were divided into ICM group (N = 571) and NIDCM group (N = 954). Compared with NIDCM, the ICM group were elder in age (65.5 ± 11.8 vs. 59.3 ± 14.4, P < 0.001) and there were more male patients (87.4% vs. 77.6%, P < 0.001). The TNT positive patients were not different between the two groups (41.3% in ICM group vs. 37.8% in NIDCM group, P = 0.215). However, the TNT expression in ICM group were significantly higher than that in NIDCM group (0.025 (0.015–0.053) vs. 0.020 (0.014–0.041), P = 0.001). As for NT-proBNP, the expression in ICM group were significantly lower than that in NIDCM group (1365.0 (515.0–3273.0) vs. 1668.0 (625.0–4238.0), P = 0.009). For echocardiography parameters, ICM patients had smaller LVDD (59.6 ± 7.6 vs. 66.0 ± 9.7, P < 0.001) and higher LVEF (40.4 ± 10.3 vs. 35.6 ± 10.9, P < 0.001). The in-hospital all-cause mortality did not show much difference between the two groups (1.1% vs. 1.9%, P = 0.204).

**Table 1:** Characteristics of included patients

	ICM (N = 571)	NIDCM (N = 954)	P value
Age (years)	65.5 ± 11.8	59.3 ± 14.4	< 0.001
Male	499 (87.4%)	740 (77.6%)	< 0.001
Smoke	214 (37.5%)	159 (16.7%)	< 0.001
Hypertension	335 (58.7%)	308 (32.3%)	< 0.001
Diabetes	271 (47.5%)	193 (20.2%)	< 0.001
PAD	22 (3.9%)	5 (0.5%)	< 0.001
COPD	8 (1.4%)	12 (1.3%)	0.812
TNT positive	236 (41.3%)	361 (37.8%)	0.215
TNT (ng/ml) (Median (IQR))	0.025 (0.015–0.053)	0.020 (0.014–0.041)	0.001
NT-proBNP (pg/ml)	1365.0 (515.0–3273.0)	1668.0 (625.0–4238.0)	0.009
CK (U/L)	76.0 (56.0–107.0)	70.0 (50.0–101.0)	0.004
CKMB (U/L)	15.0 (12.0–18.5)	14.0 (11.0–17.1)	< 0.001
Creatinine (mmol/L)	106.6 ± 66.5	105.4 ± 67.1	0.742
eGFR	71.3 ± 23.1	72.7 ± 24.3	0.272
AST (U/L)	27.8 ± 37.5	37.1 ± 108.5	0.061
ALT (U/L)	27.3 ± 45.0	49.9 ± 230.2	0.026
Hb (g/L)	132.4 ± 19.9	139.2 ± 20.6	< 0.001
PLT (*10^9/L)	200.9 ± 68.3	190.9 ± 65.3	0.004
LA (mm)	45.9 ± 6.2	48.9 ± 8.1	< 0.001
LVDD (mm)	59.6 ± 7.6	66.0 ± 9.7	< 0.001
LVEF (%)	40.4 ± 10.3	35.6 ± 10.9	< 0.001
HFrEF (EF < 40%)	302 (52.9%)	703 (73.7%)	< 0.001
All-cause mortality	6 (1.1%)	18 (1.9%)	0.204

PAD, peripheral arteria disease; COPD, chronic obstructive pulmonary disease; TNT, troponin T; IQR, interquartile range; CK, creatine kinase; CKMB, creatine kinase-MB; eGFR, estimated glomerular filtration rate; AST, aspartate aminotransferase; ALT, alanine aminotransferase; Hb, hemoglobin; PLT, platelet; LA, left atrium; LVDD, left ventricular diastolic diameter; LVEF, left ventricular ejection fraction; HFrEF, heart failure with reduced ejection fraction

### Risk factors of TNT positive

TNT values were measured at the time of admission for all the patients and 597 (39.1%) patients were positive. Logistic regressions were performed to investigate risk factors associated with TNT positive (shown in Table 2). After multivariable analyses, in ICM patients, the independent risk factors for TNT positive were age (OR 0.594, 95% CI 0.415–0.850, P = 0.004), NT-proBNP (OR 2.674, 95% CI 2.106–3.395, P < 0.001) and eGFR (OR 1.654, 95% CI 1.240–2.206, P < 0.001). In NIDCM patients, the independent risk factors were gender (OR 2.329, 95% CI 1.521–3.566, P < 0.001), PAD (OR 20.935, 95% CI 1.829–239.580, P = 0.014), NT-proBNP (OR 2.620, 95% CI 2.169–3.164, P < 0.001) and eGFR (OR 1.885, 95% CI 1.498–2.372, P < 0.001). In conclusion, NT-proBNP was independently associated with TNT in both ICM and NIDCM group.

**Table 2:** Risk factors for TNT positive

	Total patients		ICM patients		NIDCM patients	
	OR (95% CI)	P	OR (95% CI)	P	OR (95% CI)	P
NIDCM vs ICM	1.203 (0.896–1.615)	0.218				
Male	2.100 (1.478–2.984)	< 0.001		0.073	2.329 (1.521–3.566)	< 0.001
Age	0.755 (0.620–0.919)	0.005	0.594 (0.415–0.850)	0.004		0.224
Smoke	0.877 (0.645–1.192)	0.402		0.833		0.254
Hypertension	1.274 (0.973–1.670)	0.079		0.158		0.199
Diabetes	1.215 (0.911–1.622)	0.185		0.067		0.744
PAD	2.254 (0.900–5.644)	0.083		0.531	20.935 (1.829–239.580)	0.014
COPD	1.370 (0.478–3.927)	0.558		0.672		0.329
NT-proBNP	2.585 (2.236–2.998)	< 0.001	2.674 (2.106–3.395)	< 0.001	2.620 (2.169–3.164)	< 0.001
eGFR	1.788 (1.497–2.135)	< 0.001	1.654 (1.240–2.206)	< 0.001	1.885 (1.498–2.372)	< 0.001
Hb	0.871 (0.652–1.164)	0.351		0.352		0.906
HFrEF	1.135 (0.836–1.540)	0.418		0.777		0.429

PAD, peripheral arteria disease; COPD, chronic obstructive pulmonary disease; eGFR, estimated glomerular filtration rate; Hb, hemoglobin; HFrEF, heart failure with reduced ejection fraction

### Relation between TNT level and NT-proBNP level

**Fig. 1:**
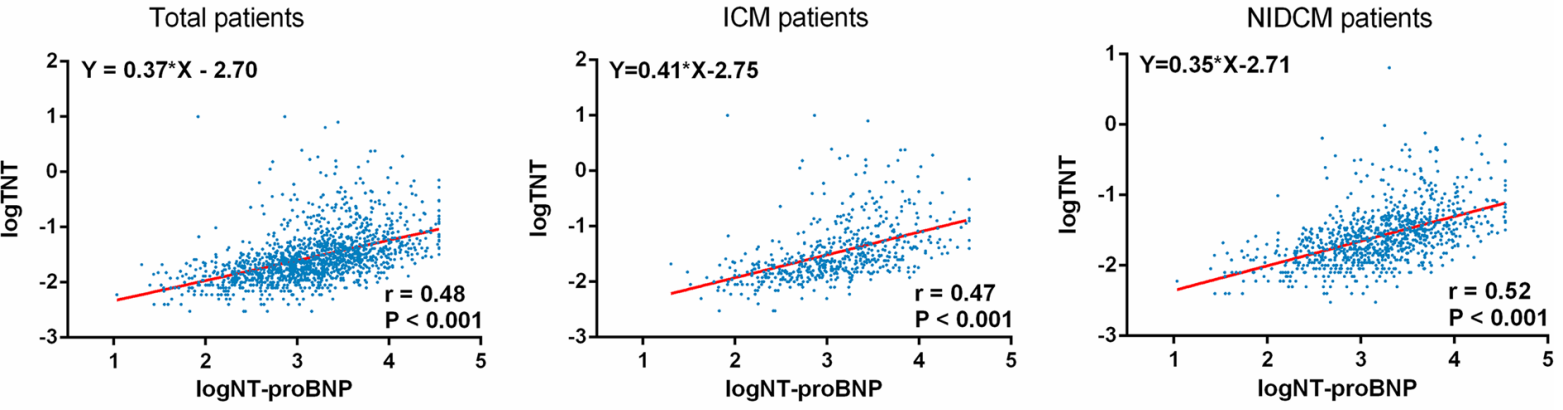
Relation between TNT and NT-proBNP level after log transformation

**Fig. 2:**
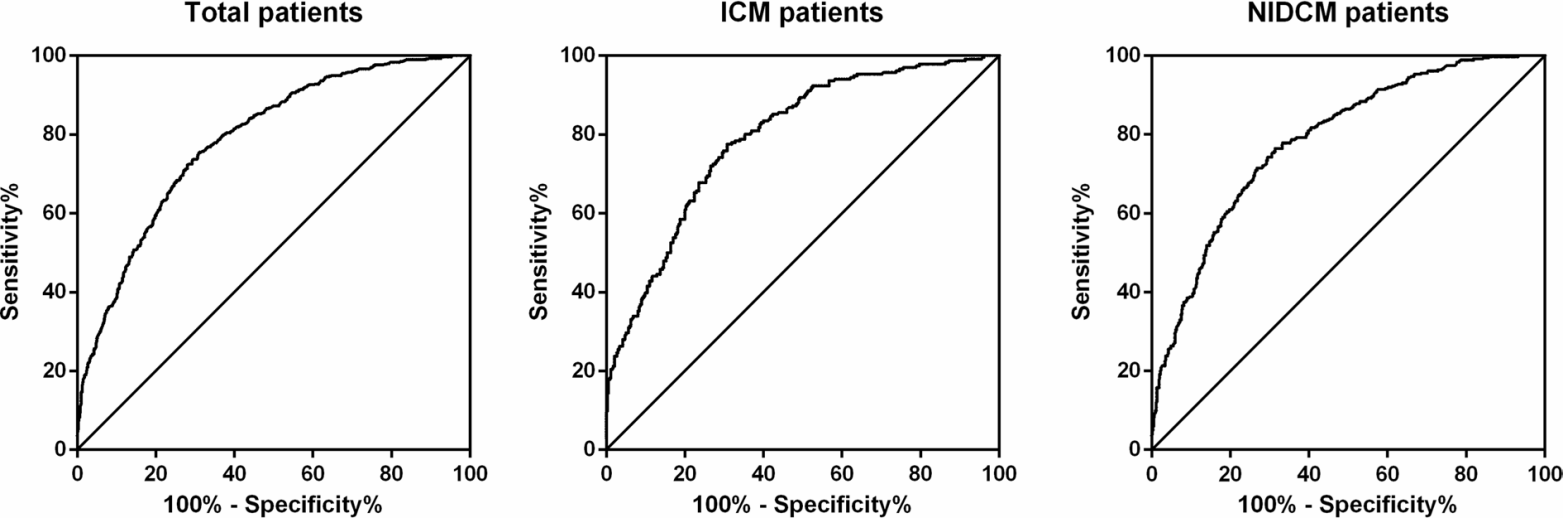
ROC curve of NT-proBNP for TNT positive. The AUC in total patients, ICM patients and NIDCM patients was 0.787, 0.793 and 0.787, respectively

After log transformation, the values of TNT and NT-proBNP were positively correlated in each group (total patients group r = 0.48, P < 0.001; ICM group r = 0.47, P < 0.001; NIDCM group r = 0.52, P < 0.001) (Fig. 1). Figure 2 showed that serum levels of NT-proBNP had moderate discriminative powers in the prediction of TNT positive. The ROC in total group, ICM group and NIDCM group were 0.787, 0.793 and 0.787, respectively.

### Risk factors of in-hospital all-cause mortality

Multiple logistic regression analysis was used to investigate risk factors of in-hospital all-cause mortality. Although the all-cause mortality was not different between ICM and NIDCM groups, the NIDCM diagnosis was associated with a lower risk of all-cause mortality after multiple adjustments (OR 0.169, 95% CI 0.040–0.718, P = 0.016). Other independent risk factors included the value of NT-proBNP (OR 8.260, 95% CI 3.168–21.533, P < 0.001), TNT (OR 8.118, 95% CI 3.205–20.562, P < 0.001), and Hb (OR 0.954, 95% CI 0.931–0.978, P < 0.001) (Table 3). In ICM and NIDCM sub-group analysis, the results were similar to that in the total patients group.

**Table 3:** Risk factors for in-hospital all-cause mortality

	Total patients		ICM patients		NIDCM patients	
	OR	P	OR	P	OR	P
NIDCM vs ICM	0.169 (0.040–0.718)	0.016	–		–	
Male	2.816 (0.691–11.474)	0.149		0.460		0.313
Age	1.011 (0.502–2.035)	0.976		0.127		0.595
Smoke	0.304 (0.304–2.718)	0.287		0.220		0.698
Hypertension	0.321 (0.097–1.062)	0.063		0.540		0.116
Diabetes	2.732 (0.899–8.304)	0.076		0.129		0.270
PAD	< 0.001	0.998		0.737		0.999
NT-proBNP	8.260 (3.168–21.533)	< 0.001	31.002 (2.203–436.197)	0.011	5.767 (1.970–16.881)	0.001
TNT	8.118 (3.205–20.562)	< 0.001	9.383 (1.472–59.819)	0.018	9.053 (2.774–29.540)	< 0.001
Hb	0.954 (0.931–0.978)	< 0.001	0.945 (0.894–0.998)	0.042	0.959 (0.932–0.987)	0.004
HFrEF	1.141 (0.851–1.141)	0.851		0.287		0.500

PAD, peripheral arteria disease; TNT, troponin T; Hb, hemoglobin; HFrEF, heart failure with reduced ejection fraction

**Fig. 3:**
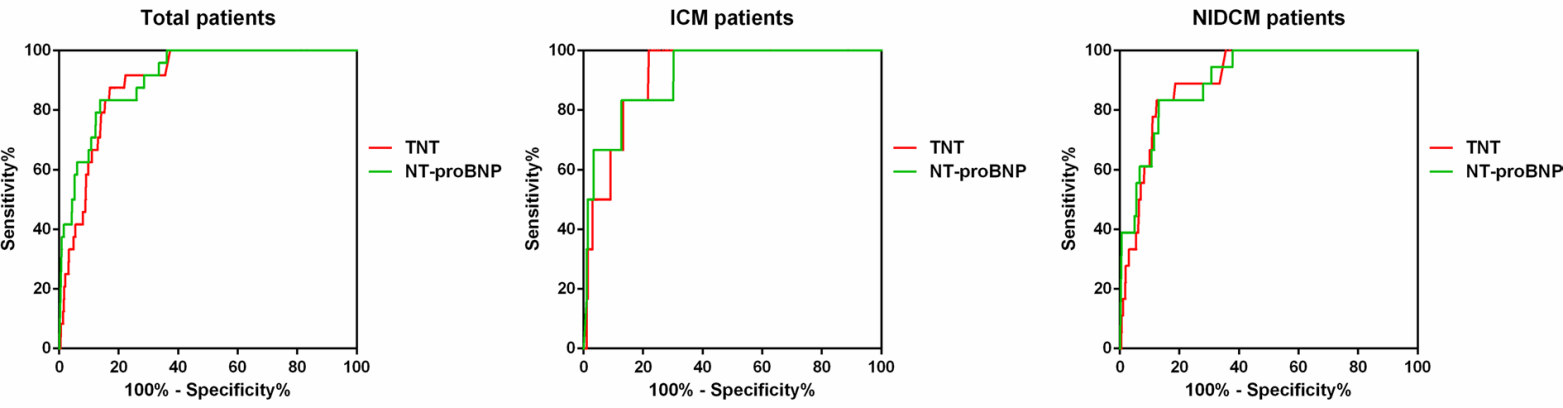
ROC curve of TNT (red line) and NT-proBNP (green line) for in-hospital all-cause mortality

As shown in Fig. 3, the predictive values of TNT and NT-proBNP for all-cause mortality were similar (Table 4). The predictive value of TNT was also similar among the groups, with AUC at 0.897, 0.917, and 0.904 in total patients, ICM and NIDCM group, respectively. However, the best cutoff values of TNT were different among these three groups. In total patients, the best cutoff value was 0.057 ng/L with the biggest sum of sensitivity and specificity (0.875 and 0.830, respectively). In ICM and NIDCM group, the best cutoff value was 0.113 ng/L and 0.048 ng/L, respectively.

**Table 4:** Area under curve and best cutoff of TNT for in-hospital all-cause mortality

	AUC (95% CI)	Best cutoff	Sensitivity (%)	Specificity (%)
*Total patients*				
TNT	0.897 (0.856–0.937)	0.057 ng/ml	87.5	83.0
NT-proBNP	0.911 (0.867–0.955)	6013 pg/ml	83.3	86.2
*ICM patients*				
TNT	0.917 (0.855–0.980)	0.113 ng/ml	83.3	86.7
NT-proBNP	0.918 (0.831–1.004)	5961 pg/ml	83.3	87.3
*NIDCM patients*				
TNT	0.904 (0.856–0.951)	0.048 ng/ml	88.9	81.4
NT-proBNP	0.906 (0.853–0.959)	6548 pg/ml	83.3	87.0

AUC, area under curve; TNT, troponin T

## Discussion

Our present study investigated the expression and prognostic value of TNT in ICM and NIDCM patients. The main findings are as following: (1) the TNT value was significantly higher in ICM group than that in NIDCM group, although TNT positive was similar between the groups; (2) NT-proBNP was independently associated with TNT and it has a good power to predict TNT positive; (3) TNT was an independent risk factor for in-hospital death and the best cutoff TNT value of predicting death was different in ICM and NIDCM group.

BNP/NT-proBNP, a type of cardiac natriuretic hormones, are released when ventricular pressure load and blood volume increase. The concentration of them reflects the severity of HF. Substantial previous studies have demonstrated that BNP/NT-proBNP levels were associated with the prognosis in HF patients for both in-hospital and long-term outcomes [9–11]. In recent years, it has been widely noticed that the abnormal release of TNT in HF patients indicates poor prognosis [12]. In 2013, guidelines recommended troponin assay as an additive tool for risk stratification in HF patients [13]. James L. and colleagues summarized the potential mechanisms of increased cardiac troponin in HF, which contained subendocardial ischemia, hypoperfusion, hypotension, inflammatory cytokine release and toxic effects of circulating neurohormones [14]. Another mechanism may be the transient elevation of left ventricular end-diastolic pressure (LVEDP). It has been confirmed that transient elevation of LVEDP can induce troponin release, apoptosis, and reversible stretch-induced stunning in the absence of ischemia [15]. The mechanism of transient elevation of LVEDP may explain why troponin and BNP or NT-proBNP are parallel expressed in previous studies [8, 16]. In our study, we also found that the level of TNT and NT-proBNP were linearly correlated.

The prognostic value of troponin has been demonstrated in nearly all different types of HF (acute vs. chronic, HF with reduced ejection fraction vs. HF with preserved ejection fraction). However, the different expression and prognostic value of troponin in acute ICM and NIDCM patients has not been fully investigated. Two previous small studies found that the expression level of troponin was higher in ICM group than that in NIDCM group [17, 18]. As far as we know, this present study was the largest one to confirm that troponin was higher in ICM than that in NIDCM. Interestingly, NT-proBNP was found to be higher in NIDCM group while TNT was lower in this group. The elevated NT-proBNP in NIDCM group was consistent with reduced LV function, such as larger LVDD and lower LVEF. This finding indicated that myocardial ischemia in ICM patients might significantly contribute to the elevation of TNT.

In clinical practice, the management of ICM patients should focus primarily on assessing whether there is an indication for revascularization. For NIDCM patients, however, it is more important to find out reversible etiology. The long-term prognosis was found to be worse in ICM than that in NIDCM [5, 6]. Our present study showed that although the in-hospital all-cause mortality did not show much difference between the two groups, the NIDCM diagnosis was associated with a lower risk of all-cause mortality after multiple adjustments. It is worth noticing that NIDCM patients showed a higher rate of HFrEF, lower left ventricular ejection fraction, more dilated left ventricle and higher NT-proBNP value. These parameters were considered to be associated with worse prognosis in HF patients, which explained why NIDCM group showed a higher absolute number of deaths. And after multiple adjustments, the etiology of NIDCM, on the contrary, were associated with a lower risk of mortality. These results indicated that NIDCM patients are more likely to have impaired cardiac function; however, with the same given parameters (such as LVEF, LVDD, NT-proBNP), NIDCM patients are less likely to experience in-hospital mortality.

Besides, identical treatments to the same comorbidities in ICM and NIDCM patients may result in different outcomes [19, 20]. Thus, it is vitally important to make early risk stratification for these two groups of patients. In a retrospective cohort study, troponin level was found to be one of the best predictors of rehospitalization after 6 months in patients with ICM [21]. Another study suggested that high serum concentration of TNT was a meaningful prognostic predictor for patients with NIDCM [22]. Our study also confirmed that TNT level was an independent factor for in-hospital all-cause mortality and the predictive value of TNT was similar in ICM and NIDCM patients. However, the best cutoff value of TNT was much higher in ICM patients than that in NIDCM patients.

Previous studies reported that about 30–70% HF patients were troponin positive when assayed in traditional methods, and up to 90–100% of the patients were troponin positive when used high sensitivity assays [23, 24]. Due to the traditional methods (threshold of TNT is 0.03 ng/mL) used in our present study, we reported a 39.1% troponin positive. The TNT positive rates were not significantly different between the two groups. In previous studies, positive or elevated troponin was associated with poor prognosis [7, 8]. However, the best cutoff value of TNT to predict mortality has not been assessed. Since quite a lot patients with HF were troponin positive, we need to define a cutoff value, which is higher than the normal level, to distinguish those patients who were really at a higher risk more effectively. In this study, we found that the best cutoff value of TNT in ICM and NIDCM group were 0.113 ng/ml and 0.048 ng/ml, respectively. This result indicated that we should take a different strategy of risk stratification using TNT in ICM and NIDCM patients. For those patients with high TNT value, sufficient communication with patients are necessary and more active treatment strategies should be adopted, such as intensive monitoring, large dose of diuretics, intravenous vasodilators, inotrope and even short-term mechanical circulatory support [3].

In conclusion, this study found that the TNT level was higher in ICM patient than that in NIDCM patients. TNT was an independent risk factor for in-hospital all-cause mortality for both ICM and NIDCM patients, although the best cutoff value was higher in ICM patients.

## Study limitations

The present study had several limitations. Firstly, this was a single-center and retrospective study in spite of relatively large sample. Secondly, we only followed the outcomes during hospitalization but long-term follow-up study will need further investigations. Last but not least, causes of heart failure are diverse, including coronary artery disease, hypertension, valve disease, cardiomyopathy and others. This study focused on ICM and NIDCM patients with dilated left ventricular. How TNT plays a role in other types of HF, such as patients with normal volumes, also needs further investigations.

## Data Availability

The datasets generated during and/or analysed during the current study are available from the corresponding author on reasonable request.
